# Supplementary material for: Splice-Junction-Based Mapping of Alternative Isoforms in the Human Proteome
Source: Cell Rep. Author manuscript; Available in PMC 2020 Jan 15. (PMC6961840; doi:10.1016/j.celrep.2019.11.026)
Supplement: 2 [file NIHMS1546469-supplement-2.zip › DF1/PXD010154/Heart/Heart_60_STK39_EENPEGPPNANEDYR.pdf]

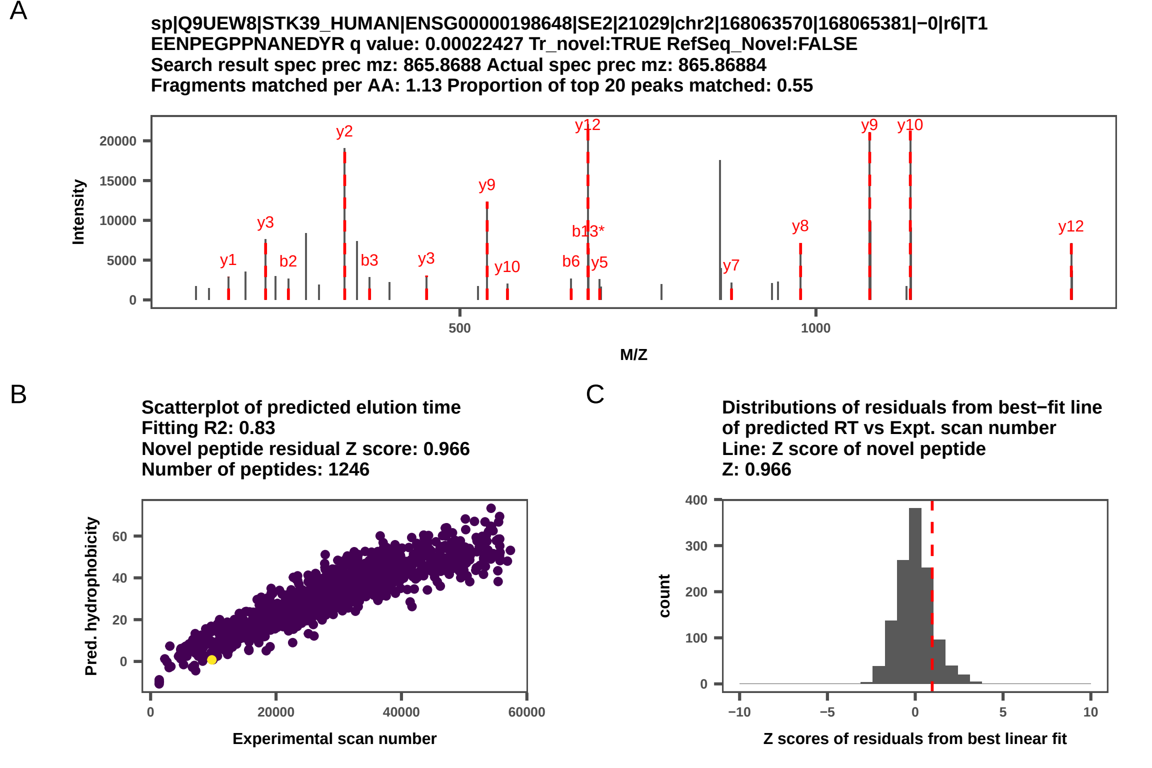
A
sp|Q9UEW8|STK39_HUMAN|ENSG00000198648|SE2|21029|chr2|168063570|168065381|−0|r6|T1
EENPEGPPNANEDYR q value: 0.00022427 Tr_novel:TRUE RefSeq_Novel:FALSE
Search result spec prec mz: 865.8688 Actual spec prec mz: 865.86884
Fragments matched per AA: 1.13 Proportion of top 20 peaks matched: 0.55
Intensity
20000
15000
10000
5000
0
y1
y3
b2
y2
b3
y3
y9
y10
b6
y12
b13*
y5
y7
y8
y9
y10
y12
500
1000
M/Z
B
Scatterplot of predicted elution time
Fitting R2: 0.83
Novel peptide residual Z score: 0.966
Number of peptides: 1246
Pred. hydrophobicity
60
40
20
0
0
20000
40000
60000
Experimental scan number
C
Distributions of residuals from best−fit line
of predicted RT vs Expt. scan number
Line: Z score of novel peptide
Z: 0.966
count
400
300
200
100
0
−10
−5
0
5
10
Z scores of residuals from best linear fit
